# Prosocial and externalizing behaviors in children raised by different-and same-gender parent families: new directions in parenting research

**DOI:** 10.3389/fpsyg.2023.1325156

**Published:** 2024-01-15

**Authors:** Roberto Baiocco, Ainzara Favini, Jessica Pistella, Nicola Carone, Anna Maria Speranza, Vittorio Lingiardi

**Affiliations:** ^1^Department of Developmental and Social Psychology, Sapienza University of Rome, Rome, Italy; ^2^Department of Humanities, University of Foggia, Foggia, Italy; ^3^Department of Systems Medicine, University of Rome Tor Vergata, Rome, Italy; ^4^Department of Dynamic and Clinical Psychology, and Health Studies, Sapienza University of Rome, Rome, Italy

**Keywords:** children adjustment, parenting practices, prosocial behaviors, externalizing behaviors, same-gender parent families

## Abstract

**Introduction:**

Limited research focused on the association between parenting practices and children’s prosocial and externalizing behaviors comparing same- and different-gender parent families. The present study considered 76 Italian families (73% same-gender and 27% different-gender parent families) with 8-year-old (SD = 2.17; 49% assigned female at birth) children born through assisted reproductive techniques, to explore parenting practices and children’s prosocial and externalizing behaviors.

**Method:**

We ran a Multiple-group-by-couple Structural Equation Model in which we estimated the predictive role of parenting on children’s behaviors, controlling for age, gender, and family socioeconomic status using the Maximum Likelihood estimation.

**Results:**

Results showed that both same- and different-gender parent families reported high levels of parental warmth and very low levels of hostility and rejection; regarding children’s behaviors, both same- and different-gender parent families reported high levels of prosociality and low levels of externalizing behaviors. In addition, same-gender parents reported significantly higher levels of children’s prosociality and parental warmth than different-gender parents. Regarding associations between parenting practices and behaviors, we found a positive association between positive parenting practices and increasing children’s prosocial behaviors and decreasing children’s externalizing behaviors, in both same- and different-gender families, controlling for family background characteristics.

**Conclusion:**

The present study encourages future research to investigate how specific parenting practices can influence behavioral adjustment in children, focusing on same-gender parent families.

## 1 Introduction

Within a socio-cognitive perspective and an interactionist view of individual functioning (e.g., Bandura et al., 2003), peoples’ behaviors, emotions, thoughts, and cognitions continuously influence and are influenced by environments and social contexts, especially at earlier developmental stages, such as childhood and early adolescence. In this perspective, family and parent–child relationships represent one of the most significant environments for positive development (Carlo et al., 2011; Lansford et al., 2018). Following the suggestions of the socioecological approach (e.g., Bronfenbrenner and Morris, 2006), the family context represents the first significant micro-system that affects children’s development. A large body of research investigated the associations between parenting practices and children’s adjustment within different-gender families that spontaneously conceived offspring (e.g., Lansford et al., 2021; Izzo et al., 2022), but limited research has considered how parent–child relationships are associated with adjustment in same-gender families or in families that utilized any medical-assisted procreation method (Baiocco et al., 2017; Golombok et al., 2018). In addition, many studies on the effects of parenting practices on individual adjustment considered preschool or early children, with limited research focused on late childhood and pre-adolescence (e.g., Ruiz-Ortiz et al., 2017).

Previous research that investigated these topics demonstrated that parent–child relationships, parenting practices, and child adjustment in families who recurred to some assisted reproduction technique substantially converge beyond the parents’ gender (Baiocco et al., 2018; Golombok et al., 2018). Of note, Italian law does not allow same-gender parents to adopt or access any form of assisted reproduction. In addition, despite the Italian health system providing assisted reproduction methods for different-gender families, the unmanageable socio-economic costs and waiting lists lead Italian families (both same and different- gender) to travel to other countries, such as Spain or the United States, to finalize the process (e.g., Shenfield et al., 2010).

### 1.1 Children’s prosocial and externalizing behaviors

Among the most salient domains of functioning for positive development in children, behavioral responses and socialization development are highly relevant (Carlo et al., 2011). On the one hand, better socio-emotional skills and adjustment in children promote the adoption of positive behaviors, such as prosocial behaviors, which involve specific behaviors oriented to other people, such as helping, taking care of others, or being kind to peers (e.g., Eisenberg et al., 2015). Prosocial behaviors play an essential role in promoting positive development in childhood and early adolescence due to their association with fewer behavioral problems (e.g., Luengo Kanacri et al., 2013; Eisenberg et al., 2015) as well as better social competence and academic performance (e.g., Caprara et al., 2014). On the other hand, issues in socio-emotional skills and self-regulative impairments increase the risk for children and adolescents to manifest problematic or adverse behavioral responses (e.g., Muris et al., 2007). These behavioral issues tap into externalizing behaviors, such as antisocial or conduct problems, aggressive tendencies, or hyperactivity. Externalizing behaviors also play a crucial role in affecting development in childhood and adolescence due to their associations with other behavioral difficulties (i.e., substance use, vandalism), emotional problems, and poor academic performance (e.g., Rapee et al., 2019).

Previous studies evidenced the importance of several individual and contextual characteristics in the emergence of adaptive or maladaptive behavioral responses in children and early adolescence, such as pre-existing risky temperamental or personality characteristics of children, adverse personality characteristics of one or both parents, family adverse experiences, parents’ psychopathology, or poor socio-emotional competences (e.g., Muris et al., 2007; Thartori et al., 2018). In line with this evidence, a growing body of research demonstrated how these two behaviors are strictly interconnected. According to previous intervention studies (e.g., Caprara et al., 2014), aggressive tendencies and prosocial behaviors are bi-directionally associated, meaning that decreases in externalizing behaviors lead to increases in prosociality and *vice-versa*. Thus, prosocial behaviors can also protect children and adolescents from maladaptive outcomes, such as academic problems, or externalizing behaviors, like verbal or physical aggression, drug use, delinquent conduct, and so on (e.g., Carlo et al., 2011). Moreover, prosocial behaviors have recently been conceptualized as the moral manifestation of the positive orientation toward others, encompassing the personality domain of resilience (Bos et al., 2023), so there are shared moral underlying mechanisms for prosociality and externalizing behaviors (e.g., Eisenberg et al., 2015; Eisner and Malti, 2015). Several previous studies attested that adjustment in children did not vary as a function of the type of couple or the type of conception method because children who lived in different-gender parent families and their same-gender counterparts showed similar levels of well-being, social competencies, and behavioral adaptation (e.g., Colpin and Soenen, 2002; Baiocco et al., 2015; Bos et al., 2023), or, in some cases, children raised by same-gender parents showed better emotional and behavioral adjustment (e.g., Lick et al., 2013).

### 1.2 Parenting practices and children’s adjustment

As stated above, a significant environment that can influence behavioral responses in childhood and early adolescence is the family context, which represents one of the most salient ones, especially at earlier stages of development (e.g., Lansford et al., 2021). Research on family functioning generally divided specific parenting practices into two main domains: positive and negative (e.g., Davis and Carlo, 2019; Di Giunta et al., 2023). Positive parenting practices, such as involvement and responsiveness, conceive all the behaviors each parent orients toward their offspring to manifest positive affection, commitment, and acceptance (Carlo et al., 2011; Lansford et al., 2021). Negative parenting practices, such as harsh parenting or control, conceive all behaviors that manifest control, limitations, aggressions, and un-involvement in children’s lives (Carlo et al., 2011; Lansford et al., 2018). Previous studies evidenced the importance of considering specific parenting practices as predictors of children’s social and behavioral adjustment rather than the macro-categories of parenting practices because considering specific strategies could account for the complexity of children’s behaviors, and they can differently influence the development of socio-emotional and moral capabilities in children (e.g., Carlo et al., 2011; Gruhn et al., 2016). In this sense, parental warmth refers to a positive parenting practice such as supporting children, caring for them, being responsive to children’s needs, and promoting open communication (Rohner, 2005; Di Giunta et al., 2023). Regarding negative parenting practices, parental hostility indicates adverse and intrusive responses to children, and coercive parenting comprises the more general dimension of over-involvement in children’s lives (Gruhn et al., 2016). On the other hand, parental rejection refers to withdrawn parental behaviors toward their children, lack of involvement and attention to their offspring’s activities, and low responsiveness to their needs (Gruhn et al., 2016).

According to the literature on different-gender families (e.g., Lansford et al., 2018; Soenens and Vansteenkiste, 2020), parent–child interactions and specific parenting practices have a substantial impact on children and early adolescents’ adjustment because they influence socio-emotional functioning, as well as self-regulation and behavioral expression (e.g., Lansford et al., 2018). Considering specific parenting practices, parental warmth has been linked to adjustment in children because it can promote an adequate development of social and relational skills, promote self-regulation, and represent a protective factor from emotional and behavioral problems (Gruhn et al., 2016; Di Giunta et al., 2023). Thus, negative parenting practices have been linked with various social, emotional, and behavioral impairments in childhood and adolescence (Di Giunta et al., 2023). Parental hostility makes children especially prone to academic problems, such as low school performance, and higher emotional problems, such as anxiety and depressive tendencies (Lansford et al., 2021). Parental rejection seems to be associated primarily with criminal, antisocial, addictive, and externalizing problems in children and adolescents (e.g., Carlo et al., 2011; Lansford et al., 2021). Therefore, previous studies attested that children who lived in families with parental warmth were better equipped to face their negative feelings and maladaptive behaviors: These children tended to be more socially skilled, so they may manifest more prosocial behaviors and less externalizing behaviors toward others (e.g., Gruhn et al., 2016). In contrast, families with recurrent negative parenting practices could predispose their children to be more prone to externalizing problems and less prosocial (Khaleque, 2013).

In terms of possible effects that the procreation method could have on family adjustment, different-gender families studies attested that, overall, there were no significant differences in terms of family and child adjustment ascribable to the conception method and that naturally conceived children and assisted-reproduction conceived children had similar developmental pathways (e.g., Colpin and Soenen, 2002). Similarly, previous studies demonstrated that associations between parenting practices and children and adolescents adjustment are substantially convergent in both different-gender and same-gender parent families, and the gender of the parent did not affect these results (e.g., Tasker, 2010; Golombok et al., 2018; Ioverno et al., 2018). Several findings demonstrated that lesbian mothers showed higher parental warmth with both their daughters and sons than mothers in different-gender parent families (e.g., Golombok et al., 2003; Fedewa et al., 2015), which tended to be warm and responsive, especially with daughters (Szkody et al., 2021). Overall, positive parenting practices have been linked with children’s adjustment (e.g., positive social behaviors), while negative parenting practices predispose children to maladaptive behaviors (e.g., aggressive or oppositional behaviors), despite the conception method was mostly not taken into account (e.g., Golombok et al., 2014, 2018; Pistella et al., 2018; Baiocco et al., 2021; Carone et al., 2021). Thus, research that focuses on these topics, which also considered possible differences between different- and same-gender families, is very limited because specific types of families were mainly analyzed, such as gay or lesbian parents (e.g., Golombok et al., 2003, 2018). However, considering that being a parent through assisted reproductive techniques is associated with peculiar feelings, routes to achieve the goal of becoming a parent, social stigma, and transitions to the new role that everyone has to face, previous literature posits that this experience could be similar in different- and same-gender families (e.g., Rubio et al., 2020).

### 1.3 The present study

The general aim of the present study was to overcome several gaps in the literature by investigating associations between positive and negative parenting practices and behavioral adjustment in children in different- and same-gender families who become parents through assisted reproduction, all living in Italy (e.g., Golombok et al., 2018; Carone et al., 2021). In particular, we analyzed concurrent associations between specific positive and negative parenting practices (i.e., parental warmth, hostility, and rejection) and specific children’s behavioral responses in terms of prosocial or externalizing behaviors (Eisner and Malti, 2015; Ruiz-Ortiz et al., 2017), in the perception of both parents within each couple considered.

First, we considered the perception of parents to identify a common view of their parenting functioning as well as of their offspring, according to previous research evidencing how children and parents substantially converge on the perception of parenting practices adopted within the family context despite the different roles that each of the two parents frequently have within the family functioning, and despite the tendency to overlook specific parenting practices of each actor (e.g., Khaleque, 2013; Yaffe, 2023). Moreover, research attested that the perception of parents on their offspring’s positive and negative behavioral responses can provide a realistic picture of the complexity of these responses in different contexts (e.g., Yaffe, 2023). Thus, due to the multi-informant nature of our constructs, we theoretically referred to the Trifactor Model (Bauer et al., 2013) by identifying a shared perspective between the two parents about parenting practices and children’s behavioral responses. To our knowledge, no previous studies examined specific parenting practices within assisted procreation methods families’ samples, and no examinations of the type of couples were previously done within this approach.

Then, examined differences in parenting behaviors and children’s behaviors across different-gender and same-gender parent families by analyzing the mean differences in the constructs mentioned above. According to previous research, we expected similar parental warmth, hostility, and rejection levels in different-gender and same-gender parent families (Tasker, 2010; Baiocco et al., 2015; Golombok et al., 2018).

Lastly, we examined the possible associations between the three parenting practices and the children’s positive and negative behaviors differently in different- and same-gender-parent families. According to previous studies (e.g., Carlo et al., 2011; Gruhn et al., 2016), we expected that parental warmth would be positively associated with children’s prosocial behaviors and negatively associated with externalizing conduct in both different-gender and same-gender parent families or that these relations would be stronger in same-gender parent families (Patterson, 2009; Fedewa et al., 2015). Following the same reasoning, we expected parental hostility and rejection to be positively associated with children’s externalizing problems and negatively related to prosocial behaviors (Lansford et al., 2021; Di Giunta et al., 2023).

We controlled all these associations for several background information, such as the child’s age and gender, as well as the family’s socioeconomic status. We considered the child’s age since our sample ranged from middle childhood to pre-adolescence, so this high variability in the age could influence associations between parenting and adjustment, according to that body of research which demonstrated how prosocial and externalizing behaviors tended to increase from middle to late childhood, and that the exercise of positive parenting practices becomes more challenging as the child grows up (e.g., Colpin and Soenen, 2002; Eisenberg et al., 2015). Similarly, we controlled for the child’s gender because previous findings evidenced how, on average, girls tended to manifest more prosocial behaviors, and boys tended to manifest more externalizing behaviors (Eisenberg et al., 2015; Eisner and Malti, 2015). Regarding family characteristics, we controlled for socioeconomic status following results which evidenced how better socioeconomic conditions foster a better family functioning, so children who lived in higher SES families on average activated more prosocial behaviors and less externalizing behaviors, and higher SES parents tended to engage in more positive parenting practices (e.g., Luengo Kanacri et al., 2013; Lansford et al., 2021).

## 2 Method

### 2.1 Procedures

The present study was drawn as a part of a wider project, designed and implemented with the general aim of investigating parenting, attachment, and child adjustment in different types of families who recurred to any kind of assisted reproduction technique (Carone et al., 2023). For couples, the inclusion criteria were: (a) lived together since the child’s birth, (b) being together at the time of the study, (c) being Italian, (d) used any kind of assisted reproduction techniques, and (e) had a child aged 6–12 years who did not suffer from any illness or disability. Recruitment was carried out through the convenience sampling technique. Most lesbian and gay parent families were recruited through “Rainbow Families” (*N* = 34, 62%)—the main 5 Italian association of sexual minority parents through assisted reproduction—with the remainder (*N* = 21, 38%) 6 recruited from the participant sample of a previous study from the research group. Heterosexual parent families were recruited through two large clinics offering assisted reproduction services in 8 Rome and Milan (*N* = 16, 76%). Also, five (24%) families were recruited through word-of-mouth from participating 9 families. See Carone et al. (2023) for more details about the study sample. Data collection was conducted between April 2021 and December 2022. The questionnaire was included as an attached document to be completed and emailed back to the researcher. Participation was voluntary and anonymous, and all provided consent to participate. Before data collection, the study was reviewed and approved by the Department of Dynamic and Clinical Psychology, and Health Studies, Sapienza University of Rome Ethics Committee (blinded for peer review; prot. n. 0000212, 24 February 2020, project title: “Same-sex and different-sex parent families through assisted reproduction: Parenting, attachment, child adjustment and neural correlates February 2020; blinded for peer review). The study was also conducted according to the guidelines of the Declaration of Helsinki.

### 2.2 Participants

The sample consisted of 76 Italian families, of which 55 were same-gender parent families (25 were two-father families, and 30 were two-mother families), and 21 were different-gender parent families (27% of the sample), for a total of 152 parents. The age of the parents ranged from 33 to 62 (M_years_ = 48.11; SD = 6.33). Regarding children, their ages ranged from 6 to 12 years old (Mean = 8.69; SD = 2.7), and, overall, their gender was equally distributed across the sample (i.e., 39 young boys −51%; 37 young girls – 49%). Families mostly lived in the North or the Center of the country (respectively, 55% of the sample in the North and 40% of the sample in the Center), and few families lived in the South (i.e., 5%). Regarding the families’ socioeconomic status, we considered the educational level of each parent within the couple, the individual income level according to Italian income stratification (Istituto Italiano di Statistica, 2020), and the work status. Overall, 32% of participating families showed a low SES (i.e., Socioeconomic Status), more than half of the families showed an average SES level (i.e., 54%), and 14% showed high SES levels (i.e., 14% of the sample). Detailed information about SES and additional background demographic information are provided in Table 1.

**Table 1:** Sample demographics.

	Total sample *N* = 76 (100%)	Different-gender parent families *N* = 21 (28%)	Two-mother families *N* = 30 (39%)	Two-father families *N* = 25 (33%)	Total same-gender parent families *N* = 55 (72%)
*Age*
Parents	48.11 (*SD* = 6.33)	*M* = 47.36 (*SD* = 6.02)	*M* = 47.13 (*SD* = 6.31)	*M* = 49.90 (*SD* = 6.34)	48.52 (*SD* = 6.29)
Child	8.69 (*SD* = 2.17)	8.50 (*SD* = 2.26)	9.00 (*SD* = 2.32)	8.48 (*SD* = 1.93)	8.77 (*SD* = 2.15)
Child Gender					
Boys	51%	43%	53%	56%	54%
Girls	49%	57%	47%	44%	45%
*Education*					
Lower education level	0%	0%	0%	0%	0%
Middle school diploma	0%	0%	0%	0%	0%
High school diploma	30%	43%	32%	18%	25%
Master/bachelor’s degree	43%	43%	43%	42%	42%
Postgraduate level	27%	14%	25%	40%	33%
*Occupation*					
Unemployed/house-worker	4%	9%	2%	4%	2%
Employee	53%	62%	58%	38%	49%
Self-employed/freelance	27%	27%	25%	28%	22%
Functionary/University professor	10%	0%	10%	18%	19%
Manager	6%	2%	5%	12%	8%
*Income*					
Extremely low (0 – 15 k €)	13%	26%	13%	2%	8%
Low (15 – 28 k €)	35%	20%	61%	18%	42%
Average (28 – 50 k €)	34%	44%	22%	36%	28%
High (50 – 100 k €)	14%	8%	2%	34%	17%
Extremely high (> 100 k €)	4%	2%	2%	10%	5%
*Relationship status*					
Engaged	0%	0%	0%	0%	0%
Cohabiting	39%	0%	70%	36%	55%
Civil union/married	61%	100%	30%	64%	45%
Other	0%	0%	0%	0%	0%

SD, Standard Deviation.

### 2.3 Measures

*Children’s prosocial and externalizing tendencies:* The Strengths and Difficulties Questionnaire (i.e., SDQ; Goodman, 1997; Tobia et al., 2013) was used to capture parents-reports of their children’s levels of prosociality and externalizing behaviors. Overall, the scale was developed to assess a variety of resources or vulnerabilities that children from 4 to 16 years old can behaviorally manifest, and it originally included five specific subscales: Emotional Symptoms, Behavioral/Externalizing Symptoms, Attention or Hyperactivity Problems, Peer Problems, and Prosocial Behaviors. For the present study, four items for prosocial behaviors (i.e., “Helpful if someone is hurt, upset, or feeling ill,” or “Considerate of other people’s feelings”), and four items for externalizing behaviors (i.e., “Often fights with other children or bullies them,” or “Often lies or cheats”) were considered, in the parent-report version (Tobia et al., 2013). Each parent rated items on a 3-point Likert scale, ranging from 0 “not true” to 2 “absolutely true.” Previous studies supported the instrument’s structure and psychometric properties in Italy (e.g., Tobia et al., 2013). In our study, reliability was good (Cronbach’ α for prosocial behaviors = 0.80; Cronbach’ α for externalizing behaviors = 0.75). Further information is provided in Supplementary Table S1.

*Parenting behaviors:* The Parental Acceptance and Rejection/Control Questionnaire (PARQ; Rohner, 2005) was used to assess parental behaviors regarding their children. In particular, following previous studies (e.g., Bornstein et al., 2015), for the positive parenting strategies, we considered the dimension of parental Warmth, which conceives parental acceptance, as well as verbal and non-verbal emotional relationships toward children, while for measures of negative parenting practices, we considered parental Hostility (i.e., which conceives parental aggressions, verbal or physical behaviors, that results of disapproving children), and parental Rejection (i.e., which conceives parental indifference, and criticism toward children). The original version of the scale comprises 60 items assessing specific parenting strategies. For the present study, we “*ad hoc*” selected 7 items from the short version of the questionnaire for the domain of Warmth, 4 items for the domain of Hostility, and 3 items for the Rejection domain (Rohner, 2005). Items were rated on a four-point Likert scale, ranging from 1 “never or almost never” to 4 “every day.” Previous studies supported this questionnaire’s structure and psychometric properties across cultures (e.g., Bornstein et al., 2015). In our study, the internal consistency of the sub-scales was acceptable, ranging from Cronbach’ α = 0.64 for the Rejection subscale to Cronbach’ α = 0.78 for the Warmth subscale. Further information is provided in Supplementary Table S1.

### 2.4 Statistical analyses

We followed a statistical procedure divided into several steps to answer our research questions. Preliminary, considering that our data were cross-informant reports about parents’ parenting behaviors toward their children as well as their children’s behavioral tendencies, we moved into a Trifactor Framework to identify underlying latent variables for each dimension, which reflects the familiar perspective of two different informants on the same construct, identifying means for each construct (Bauer et al., 2013). For doing this, we estimated a common latent factor of each construct by constraining items to be equal across the two informants in terms of factor loadings (i.e., metric structure) and intercepts (i.e., scalar structure). The first item of each latent factor was fixed to 0, and the variance of the latent factor was scaled to 1, with a mean of 0, for model identification (Bauer et al., 2013).

Once multi-informant constructs were identified, we descriptively examined mean differences in children’s behaviors and parenting behaviors across different types of families (i.e., same-gender parents and different-gender parents), referring to the Analysis of Variance framework. Lastly, to investigate the possible predictive effects of the three parenting strategies considered (i.e., Warmth, Hostility, and Rejection) on children’s prosocial and externalizing behaviors, we ran a Multiple-group-by-couple Structural Equation Model in which we estimated the predictive role of parenting on child’s behaviors, taking into account the possible effects of child and family background characteristics.

We estimated each model using the Maximum Likelihood estimation with Robust standard errors (MLR) estimator to account for sub-group differences parsimoniously (Byrne, 2013), and several criteria were considered as indices of good or adequate model fit: (a) *χ*2 difference test model comparison (Kline, 2010); (b) Comparative Fit Index (CFI) values higher than 0.90 (Kline, 2010); (c) Root-Mean-Square Error of Approximation (RMSEA) and its confidence intervals and *p* values <0.08 as indicators of reasonable fit (Kenny et al., 2015); (d) ΔCFI and ΔRMSEA lower than 0.10 (Hooper et al., 2008).

## 3 Results

### 3.1 Identification of multi-informant constructs of children’s behaviors and parenting dimensions

First, we identified latent variables of multi-informant constructs, isolating what each parent’s perspective had in common with the other parent’s perspective regarding their children’s behaviors and their own parenting practices. Thus, we ran five different trifactor models to test the structure of each variable (i.e., Parental Warmth, Parental Hostility, Parental Rejection, Prosocial Behaviors, and Externalizing Behaviors; Bauer et al., 2013). The results of this procedure are summarized in Table 2.

**Table 2:** Multi-informant constructs of the study variables: summary of goodness-of-fit statistics.

	*χ* ^2^	*Df*	Scal. Corr.	CFI	TLI	RMSEA
*Children Behaviors*
Prosocial behaviors multi-informant model	28.05	22	0.948	0.93	0.86	0.08 (0.00–0.17)
Externalizing behaviors multi-informant model	13.97*	6	0.947	0.94	0.84	0.12 (0.04–0.22)
*Parenting behaviors*
Warmth multi-informant model	44.74	44	1.355	0.96	0.95	0.03 (0.00–0.08)
Hostility multi-informant model	20.20*	11	1.075	0.93	0.81	0.10 (0.01–0.17)
Rejection multi-informant model	4.03	4	1.175	0.99	0.99	0.01 (0.00–0.17)

*χ*^2^ = Chi-square Goodness of Fit; Df = degrees of freedom; Scal. Corr. = Scaling Correction Factor; CFI=Comparative Fit Index; TLI = Tucker-Lewis comparative fit Index; RMSEA = Root-Mean-Square Error of Approximation. All Δ index comparisons were made comparing the model with the previous one. *p < 0.05, **p < 0.01, ***p < 0.001.

For what concerns children’s behaviors, the multi-informant model for prosocial behavior showed an acceptable fit [*χ*^2^ (22) = 28.05, *p* = 0.17 (n.s.), RMSEA = 0.08 (0.00–0.17), CFI = 0.93], despite the high value of the upper C. I. of the RMSEA, which could attest some kind of model misspecifications (e.g., Hooper et al., 2008). The multi-informant model for externalizing behaviors showed a quite poor fit [*χ*^2^ (6) = 13.97, *p* = 0.03 (< 0.05), RMSEA = 0.12 (0.04–0.22), CFI = 0.94], due to a *χ*^2^ significance value of p, as well as very high RMSEA value and C.I.s intervals (e.g., Hooper et al., 2008). Regarding the parenting practices models, the Warmth [*χ*^2^ (44) = 44.74, *p* = 0.29 (n.s.), RMSEA = 0.03 (0.00–0.08), CFI = 0.96] and the Rejection [*χ*^2^ (4) = 4.03, *p* = 0.40 (n.s.), RMSEA = 0.01 (0.00–0.17), CFI = 0.99] models demonstrated adequate fits, despite the high value of the upper C. I. of the RMSEA, while the Hostility model [*χ*^2^ (11) = 20.20, *p* = 0.04 (< 0.05), RMSEA = 0.10 (0.00–0.17), CFI = 0.93] showed a poor fit, by referring to the significant *χ*^2^ value of p and the high RMSEA values and C.I.s (e.g., Hooper et al., 2008).

### 3.2 Mean differences in children’s behaviors and parenting dimensions

**Figure 1:**
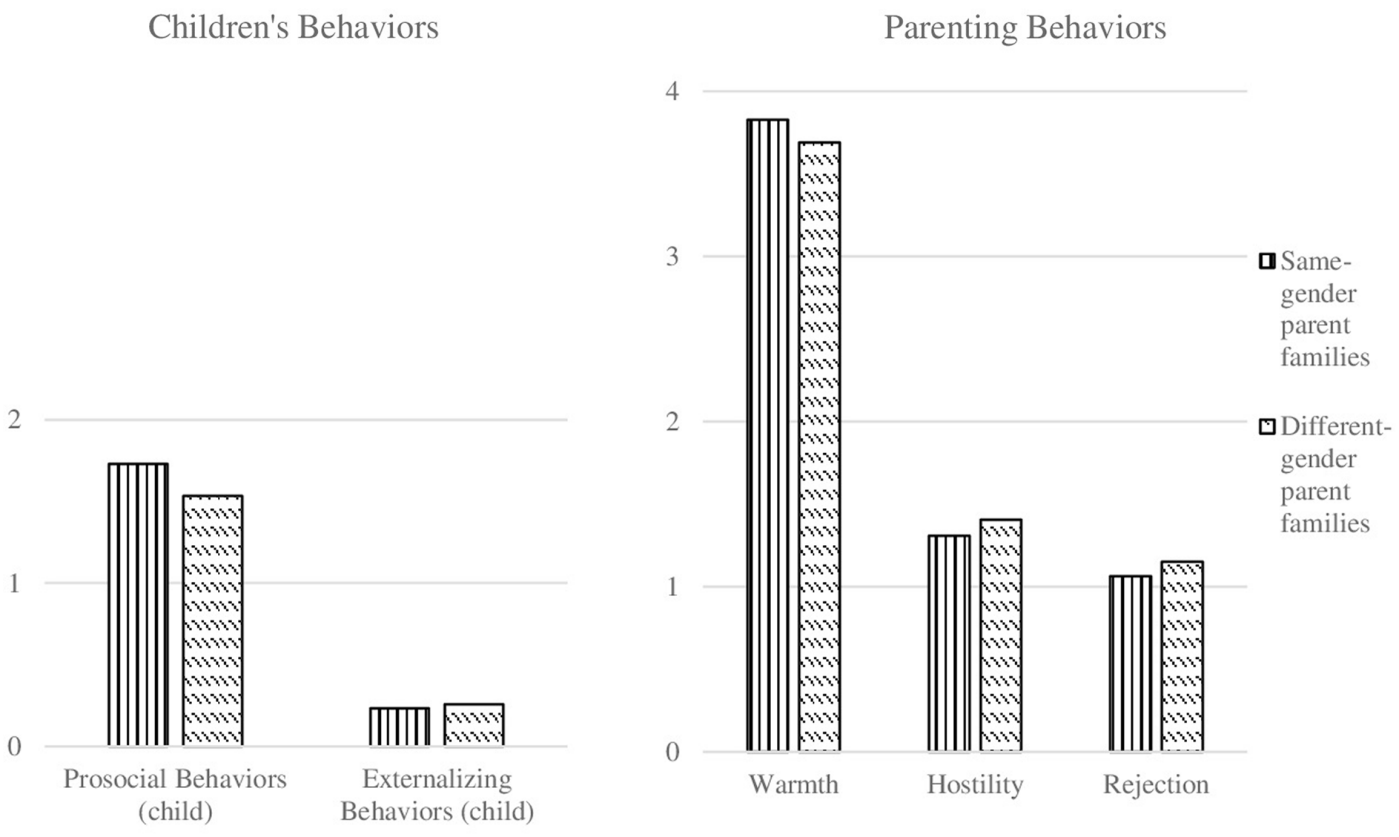
Mean differences in childrens behaviors and parenting practices.

To investigate mean differences in children’s behavioral tendencies and parenting dimensions across different types of families (i.e., different-gender parent families and same-gender parent families), we ran a series of ANOVAs (see Table 3). As regards children’s behavioral tendencies, our models revealed a significant difference in prosocial behaviors with a strong effect size [*F* (1,74) = 6.61; *p* = 0.01 (< 0.05); *η*^2^ = 0.08] and no significant differences in externalizing behaviors [*F* (1,74) = 0.14; *p* = 0.71 (n.s.); *η*^2^ = 0.00], meaning that children of same-gender parent families showed significantly more prosociality than their counterpart that lived in different-gender parent families (respectively, Prosocial behavior Mean_SS_ = 1.73; Prosocial behavior Mean_HS_ = 1.53). As regards parenting behaviors, our models attested significant differences in parents’ levels of Warmth with a medium effect size [*F* (1,74) = 4.42; *p* = 0.04 (< 0.05); *η*^2^ = 0.06], and quite similar levels in parental Hostility [*F* (1,74) = 1.12; *p* = 0.29 (n.s.); *η*^2^ = 0.01], and Rejection [*F* (1,74) = 2.32; *p* = 0.13 (n.s.); *η*^2^ = 0.03], meaning that in same-gender parent families, parents showed significantly higher levels of Warmth than their different-gender counterparts toward their children (respectively, Warmth Mean_SS_ = 3.83; Warmth Mean_HS_ = 3.60), but similar levels of Hostility and Rejection strategies. The results of this step are shown in Figure 1.

**Table 3:** Mean differences in children’s behaviors and parenting dimensions.

	Type of family	Mean (SD)	F (*df*)	P	η^2^
*Children’s behaviors*					
Prosocial behaviors	Different-gender	1.53 (0.38)	6.61 (1,74)	0.01	0.08
	Same-gender	1.73 (0.26)			
Externalizing behaviors	Different-gender	0.26 (0.29)	0.14 (1,74)	0.71	0.00
	Same-gender	0.23 (0.24)			
*Parenting practices*					
Warmth	Different-gender	3.60 (0.31)	4.42 (1,74)	0.03	0.06
	Same-gender	3.83 (0.22)			
Hostility	Different-gender	1.40 (0.40)	1.12 (1,74)	0.29	0.01
	Same-gender	1.31 (0.33)			
Rejection	Different-gender	1.15 (0.32)	2.32 (1,74)	0.13	0.03
	Same-gender	1.06 (0.17)			

SD, Standard Deviation; df, Degrees of Freedom; η^2^, eta square.

### 3.3 Relations between parenting and prosocial and externalizing behaviors

**Figure 2:**
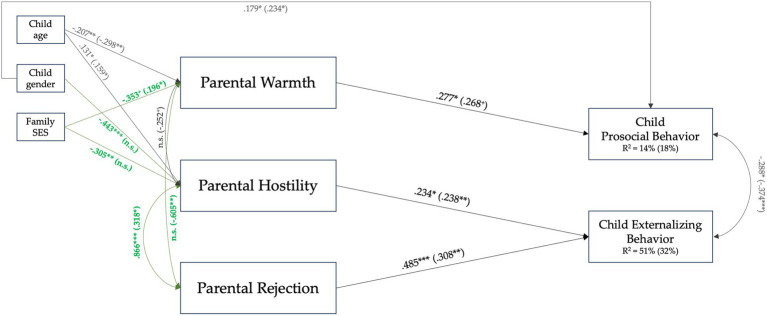
Multi-group by family types SEM model. Model fit: χ^2^ (23) = 26.44 (n.s.); CFI = 0.98; TLI = 0.95; RMSEA = 0.06 (0.00.15); SRMR = 0.08. The first value refers to different-gender parents, while the second refers to same-gender parents. Green bolded lines and values represent parameters that were freely estimated. *p < 0.060; * *p* < 0.050; ** *p* < 0.010; *** *p* < 0.001.

As the last step, we investigated whether specific positive and negative parenting practices could predict children’s behavioral responses differently in different-gender and same-gender parent families, controlling for the child’s and the family’s background characteristics. In doing so, we concurrently estimated the same SEM model into the two different groups of family typology (i.e., a multi-group approach) to examine any possible differences in predicting parenting practices on child behaviors for different-gender and same-gender parent families (Figure 2).

We compared a model in which all the parameters were freely estimated with a model with all the parameters fixed to be equal across groups adopting the strategy evidenced in the analytic plan section by also referring to Modification Indices as additional cues to release parameters (Steenkamp and Baumgartner, 1998). We released one parameter per time, comparing each nested model with the previous one, until we reached a non-significant increase in the χ^2^. The results of this procedure are summarized in Table 4.

**Table 4:** Path model of parenting practices in children’s behavioral tendencies in different-gender and same-gender parent families: summary of goodness-of-fit statistics.

							Model Comparison			
	*χ* ^2^	*Df*	Scal. Corr.	CFI	RMSEA		*χ*^2^ diff	Δ D*f*	Δ CFI	Δ RMSEA
Model Different-gender parents	1.46	3	1.011	1.00	0.00 (0.00–0.27)					
Model Same-gender parents	0.63	3	0.639	1.00	0.00 (0.00–0.10)					
Model a: All free parameters	2.71	6	0.826	1.00	0.00 (0.00–0.11)					
Model b: All fixed	72.09	28	0.764	0.69	0.20 (0.14–0.26)	b vs. a	70.74***	22	−0.31	0.20
Model c: Partial fixed	26.44	23	0.794	0.98	0.06 (0.00–0.15)	c vs. a	23.97	17	−0.02	0.06

*χ*^2^ = Chi-square Goodness of Fit; Df = degrees of freedom; Scal. Corr. = Scaling Correction Factor; CFI=Comparative Fit Index; RMSEA = Root-Mean-Square Error of Approximation. All Δ index comparisons were made comparing the model with the previous one. *p < 0.05; **p < 0.01; ***p < 0.001.

The unconstrained model (i.e., Model a, Table 3) fits the data well [*χ*^2^ (6) = 2.71, *p* = 0.845 (n.s.), RMSEA = 0.00 (0.00–0.11), CFI = 1.00], despite the high value of the upper C. I. of the RMSEA, which could represent some misspecification of the model (e.g., Hooper et al., 2008). When we tested a model with all the parameters fixed to be equal across the two groups, the model fit was significantly worse [*χ*^2^ (28) = 72.10, *p* = 0.000 (< 0.001), RMSEA = 0.20 (0.15–0.26), CFI = 0.69], suggesting that some parameters could significantly differ between different-gender and same-gender parent families’ groups. In the final partially constrained model (Model c, Table 3), we released the correlation between Hostility and Rejection, the effect of SES and children’s gender on Hostility, the effects of SES on Warmth, and the correlation between Rejection and Warmth [*χ*^2^ (23) = 26.44, *p* = 0.280 (n.s.), RMSEA = 0.06 (0.00–0.15), CFI = 0.98].

The findings of this model showed several significant associations: associations among parenting practices and children’s behavioral responses regarding prosociality and externalizing tendencies did not differ from same-gender parent families to different-gender families. In both types of families, higher parental hostility and rejection of parents were associated with higher externalizing behaviors in their children, controlling for children’s gender, age, and family socioeconomic status. In addition, higher parental warmth was associated with higher prosocial behaviors in children of different-gender parent families and tended to be significant in same-gender parent families (*p* < 0.06).

## 4 Discussion

The present study aimed to investigate, from a shared parent’s point of view (i.e., by considering what in their perception of family functioning was in common; Bauer et al., 2013), concurrent associations between specific positive (warmth) and negative (hostility and rejection) parenting practices and behavioral adjustment (prosocial and externalizing behaviors) in children from same-gender and different-gender families recurring to assisted procreation method to become parents (Carone et al., 2021; Shenkman et al., 2023). Given the considerable increase in recurring assisted reproduction methods not only by same-gender families but also by different-gender families in Italy (e.g., Baiocco et al., 2018; Shenkman et al., 2023), it is crucial to examine associations between children’s adjustment and parenting practices in these contexts (Baiocco et al., 2018), to expand the knowledge on these topics that came from traditional research on family functioning in different-gender families who become parents naturally (e.g., Lansford et al., 2021).

Our findings evidenced that, according to the Trifactor Model (Bauer et al., 2013), it was possible to combine different perspectives of parents on their parenting practices and their offspring’s behaviors into several common underlying constructs, which reflect the shared parenting practices within the parent dyad, as well as a unified view of children’s behavioral adjustment (e.g., Basili et al., 2021; Yaffe, 2023). In particular, we identified a common view of parental warmth and rejection that parents tended to adopt regarding their children. In contrast, the model in which we combined the shared parental perception regarding parental hostility demonstrated a quite poor fit (Kenny et al., 2015). We reasoned that the specific sub-domain of parental hostility taps into the more general dimension of parental rejection, but contrary to the practice of rejection, parental hostility encompasses more severe forms of negative parenting, such as the manifestation of aggressive behaviors toward children. This parenting practice is less adopted in the general population, and therefore it could be more challenging to identify a shared view between the two parents (Putnick, 2019). Moreover, as demonstrated for other negative constructs, individuals tend to under-report maladaptive behaviors or parenting practices, according to the social desirability bias (De Los Reyes, 2011; Putnick, 2019). Regarding children’s behavioral responses, we identified a shared parental perception of their offspring’s prosocial behaviors, but the model for externalizing behaviors demonstrated a quite poor fit, similar to what we found for the hostility model (Kenny et al., 2015). The parallelism between these results could be ascribable to similar reasonings because, as for the reports for parenting practices, parents may under-report their children’s externalizing behaviors, which may result in a more difficult identification of underlying common view of externalizing behaviors of their children (De Los Reyes, 2011).

Regarding levels of children’s behavioral adjustment and parenting practices used, at mean levels, we confirmed similar behavioral patterns within the family contexts in both same-gender and different-gender parent families. In particular, considering parenting practices, we found high overall levels of parental warmth in both families and higher levels of warmth in same-gender parent families compared with their different-gender counterparts. Additionally, we confirmed a similar lower adoption of parental hostility and rejection in these two types of families (e.g., Ioverno et al., 2018; Carone et al., 2021). These results are in line with previous studies which evidenced similar parenting styles and practices and parent–child relationships in different types of families (e.g., Golombok et al., 2014, 2018; Ioverno et al., 2018; Shenkman et al., 2023), confirming that, for what concerns positive parenting practices, same-gender parents tended to be more reactive and warmth toward their children (e.g., Fedewa et al., 2015). Regarding children’s behavioral responses, we did not find differences between same-gender and different-gender parents in their perception of children’s externalizing behaviors, which were very low. At the same time, we found very high levels of prosocial behaviors in both types of families. Between different- and same-gender parents, there was a significant difference in terms of mean levels of prosocial behaviors, as same-gender parents reported significantly higher prosociality of their children than different-gender parents. Similarly, we found no differences between same-gender and different-gender parents in their perception of negative parenting practices adopted by their children. However, our results attested a moderate difference in parental warmth mean levels between these two types of families, as same-gender parents reported significantly higher warmth than their different-gender counterparts.

Overall, these findings are in line with previous studies that highlighted how children’s behavioral adjustment, as well as parental functioning, tended to be similar across different types of families, even when comparing same-gender and different-gender parent families, which frequently showed no significant differences in their family functioning and behavioral adjustment (Tasker, 2010; Golombok et al., 2018). Thus, our findings that showed higher parental warmth and higher prosocial behaviors in same-gender parent families compared with their different-gender counterparts confirmed previous research, which emphasized how, in some cases, same-gender parent families showed a better adjustment in terms of parenting functioning and children’s behavioral responses than different-gender parent families (e.g., Bos et al., 2016; Baiocco et al., 2018). This difference could be read in the light of the higher involvement and commitment that same-gender parents tended to have in their offspring’s development as a result of their higher perceived social stigma, so they could foster in their children higher empathy-related responding, which, in turn, may predispose them to enact more prosocial behaviors (Eisner and Malti, 2015; Baiocco et al., 2018).

Regarding the predictions of parenting practices on children’s behavioral adjustment, associations between specific parenting practices and specific behavioral responses showed quite similar patterns among same-gender and different-gender parent families, and we did not find differences in these associations ascribable to the gender of the parents (e.g., Carone et al., 2021; Shenkman et al., 2023). In both same-gender and different-gender families, hostility and rejection enacted by both parents were significantly associated with higher externalizing behaviors in children. Patterns of adaptive parent–child relationships evidenced similar findings: in both same-gender and different-gender parent families, parental warmth shown by parents was associated with prosocial behaviors in children. These results hold controlling for the child’s age, gender, and the family’s socioeconomic status. Thus, parents who express warmth behaviors toward their children can contribute to developing a positive family context that, in turn, promotes adequate socio-emotional development in children, who become better equipped in terms of empathy-related responding behaviors, which are more inclined to manifest positive behaviors toward others, such as helping others, sharing with others their things, and support others (Carlo et al., 2011; Gruhn et al., 2016), as we also found at mean-level. On the other hand, parents who frequently adopt negative parenting practices, such as rejection or hostility toward their offspring, increase the conflicts within the family context, which negatively affects socio-emotional development in their children because they tend to experience feelings of non-acceptance and not validation from their parents (e.g., Lansford et al., 2021; Folker et al., 2023). This conflictual family context, in turn, could increase the risk of developing behavioral and/or emotional problems, such as delinquent, antisocial, or aggressive behaviors (Lansford et al., 2018, 2021).

In terms of combined associations between negative parenting practices and positive behaviors, parental warmth did not affect externalizing behaviors, and parental hostility and rejection did not affect the manifestation of prosociality, so our findings did not replicate findings of previous different-gender family studies (e.g., Di Giunta et al., 2023; Folker et al., 2023). We wondered about this partial incongruence and identified several possible explanations. For one, previous studies mainly considered the parents’ contribution separately, only the contribution of mothers, and considered only different-gender parent families (Carlo et al., 2011; Lansford et al., 2018; Di Giunta et al., 2023). Differently from this previous body of studies, we adopted a different multi-informant strategy, which captured what the two parents of any gender within the couple shared in common in terms of perception of their offspring and their own behaviors (e.g., Baiocco et al., 2018). Moreover, our sample was slightly small, so further research should capture these associations on a broader sample of different types of families to test whether the protective role of warmth on externalizing behavior, as well as the vulnerability role of hostility and rejection for prosocial behaviors, should be replicated (Carlo et al., 2011; Folker et al., 2023).

## 5 Limitations and future directions

Despite the strengths of the present study, there are several limitations. First, our sample size was quite small (Hooper et al., 2008; Kenny et al., 2015). Future research should test the same associations between positive and negative parenting practices on children’s behavioral responses in same-gender and different-gender parent families within more comprehensive samples to better investigate these associations. Also from a statistical point of view, our sample was small too, so future research could integrate in their design a power analysis to set the minimum number of subjects that should be involved in research to adequately estimate the effects between the study variables (Wolf et al., 2013). Second, although we aimed to identify causal associations between positive and negative parenting practices on children’s adjustment, we tested them within a cross-sectional design, which may fail to find real causal associations among the constructs (Hooper et al., 2008). Therefore, future family research could benefit from including different assessment waves to better capture the predictive role of parenting within different types of families on children’s adjustment. Third, in our study, we considered only the perception of parents on their parenting behaviors and their offspring’s behavioral adjustment due to the unavailability of children reports and models for children’s externalizing behaviors and parental Hostility practices evidenced low quality in their fits, probably due to the lower number of subjects in the sample and the limited number of degrees of freedom (e.g., Kenny et al., 2015). Children’s reports could lead to reporter biases, such as social desirability, especially in the case of offspring behaviors, which are extremely sensitive outcomes of children’s general adjustment and desirable behaviors in broader social contexts. Thus, future studies could include other reporters for children’s behaviors, such as their teachers, providing a picture of children’s adjustment in the school context, which is another crucial social context for children’s and adolescents’ development (e.g., Luengo Kanacri et al., 2013; Caprara et al., 2014). Longitudinal and cross-cultural research is needed to understand better the relevance of positive or negative parental practices on the adjustment of children raised in same-gender parent families across different developmental periods. Moreover, future research should investigate the potential role of bullying, microaggressions, and inequalities experienced by children regarding their non-traditional family composition (Carone et al., 2021). Accordingly, future studies should explore the link between parenting behaviors and children’s well-being in other sexual and gender minority parents (e.g., queer, pansexual, or transgender parent families).

Despite its limitations, the present study is a step forward in the field of research that investigates how children’s adjustment is influenced by familiar variables, such as specific parenting practices or family processes (e.g., Eisner and Malti, 2015; Thartori et al., 2018), and underlined the importance to analyze these topics within different types of families, such as same-gender parent families, also considering families who recurred to alternative procreation techniques (Rubio et al., 2020). We supported how, beyond the gender of the parents and the procreation method, adopting positive parenting strategies, such as being a warm and committed parent, predisposes children to better socio-emotional functioning, leading to the exercise of more prosocial behaviors (e.g., Baiocco et al., 2015; Eisner and Malti, 2015; Lansford et al., 2021). Our findings suggest the relevance of positive parenting practices for understanding subjective well-being and positive behavior development among children, regardless of parents’ gender and sexual orientation (Bos et al., 2023). Overall, societal-level actions (e.g., interventions to promote positive attitudes toward same-gender parent families) can be essential to build a more inclusive and safe society for all family compositions, regardless of parents’ sexual orientation or gender identity (Patterson, 2009). Thus, societal projects could benefit by including in their strategies specific actions to support and empower all the types of families who recurred to assisted reproductive techniques the adoption of positive parenting practices to sustain and foster their children’s adaptation (e.g., Rubio et al., 2020).

## Data availability statement

The raw data supporting the conclusions of this article will be made available by the authors, without undue reservation.

## Ethics statement

The studies involving humans were approved by Department of Dynamic and Clinical Psychology, and Health Studies, Sapienza University of Rome, Italy. The studies were conducted in accordance with the local legislation and institutional requirements. The participants provided their written informed consent to participate in this study.

## Author contributions

RB, NC, AMS, and VL contributed to the study’s conception and design. RB, AF, NC, and VL performed material preparation, data collection, and analysis. RB, AF, and JP wrote the first draft of the manuscript. All authors commented on previous versions of the manuscript. All authors read and approved the final manuscript.
