# Supplementary material for: Prosocial and externalizing behaviors in children raised by different-and same-gender parent families: new directions in parenting research
Source: Front Psychol. 2024 Jan 15;14:1325156. doi: 10.3389/fpsyg.2023.1325156 (PMC10825949; doi:10.3389/fpsyg.2023.1325156)
Supplement: Supplementary file 1 [file Table_1.DOCX]

**Table S1. Descriptive statistics and correlations among the study variables**

|  | (1) | (2) | (3) | (4) | (5) | (6) | (7) | (8) |
| --- | --- | --- | --- | --- | --- | --- | --- | --- |
| Child Gender (1) | 1 |  |  |  |  |  |  |  |
| Child Age (2) | -.21 | 1 |  |  |  |  |  |  |
| Family SES (3) | -.14 | -.03 | 1 |  |  |  |  |  |
| Warmth (4) | .09 | -.22 | .08 | 1 |  |  |  |  |
| Hostility (5) | -.18 | .10 | .07 | -.22 | 1 |  |  |  |
| Rejection (6) | -.05 | -.01 | .02 | -.32^**^ | .52^***^ | 1 |  |  |
| Prosocial Behaviors (7) | .25^*^ | -.17 | .01 | .36^**^ | -.10 | -.17 | 1 |  |
| Externalizing Behaviors (8) | .06 | -.01 | -.21 | -.36** | .39*** | .44*** | -.20 | 1 |
|  |  |  |  |  |  |  |  |  |
| Mean | - | - | - | 3.79 | 1.33 | 1.09 | 1.63 | .35 |
| St. Dev. | - | - | - | .26 | .35 | .22 | .34 | .30 |
| Cronbach’s α | - | - | - | .78 | .69 | .64 | .80 | .75 |

*Notes*: SES = Socioeconomic status; St. Dev. = Standard Deviation.

* *p* < .05, ** *p* < .01, *** *p* < .001.
